# Gut–Heart Axis in HFpEF: The Emerging Role of Microbiome-Driven Inflammation and Endothelial Dysfunction

**DOI:** 10.3390/biom16030401

**Published:** 2026-03-08

**Authors:** Sheeza Nawaz, Tadahisa Sugiura, Ismaila Yusuf, Abdullah Sultany

**Affiliations:** 1 Department of Medicine, Robert Packer Hospital Guthrie, Pennsylvania, PA 18840, USA; sheeza.nawaz@guthrie.org (S.N.); ismaila.yusuf@guthrie.org (I.Y.); abdullah.sultany@guthrie.org (A.S.); 2 Department of Cardiothoracic and Vascular Surgery, Montefiore Medical Center, Albert Einstein College of Medicine, New York, NY 10467, USA

**Keywords:** heart failure with preserved ejection fraction, gut microbiota, microbiome-driven inflammation, endothelial dysfunction, TMAO, short-chain fatty acids, precision medicine

## Abstract

Heart failure with preserved ejection fraction (HFpEF) represents the predominant form of heart failure, affecting over 50% of all heart failure patients with increasing prevalence in aging populations. Despite significant advances in cardiovascular medicine, HFpEF remains a complex clinical syndrome with poorly understood pathophysiology and limited treatment options. While most studies have traditionally focused on the renin–angiotensin–aldosterone system (RAAS) and other related mechanisms, emerging evidence has unveiled a critical bidirectional relationship between dysregulation of gut microbiota and HFpEF development. This phenomenon, mediated through microbiome-driven inflammation and endothelial dysfunction, introduces a novel concept and potential emerging conceptual framework in understanding HFpEF. This comprehensive review explores this novel gut–heart axis by synthesizing the latest evidence from original studies and clinical trials. We discuss novel mechanisms involving bacterial metabolites, including short-chain fatty acids (SCFAs), trimethylamine N-oxide (TMAO), bile acids, and amino acid derivatives. We also examine how gut dysbiosis may contribute to systemic inflammation through lipopolysaccharide translocation, NLRP3 inflammasome activation, and endothelial dysfunction. Furthermore, clinical trials investigating microbiome-targeted interventions, including probiotics, fecal microbiota transplantation, metabolite supplementation, and precision medicine approaches, are critically evaluated for their therapeutic potential. This review provides a framework for hypothesis generation and future research directions about therapeutic strategies targeting the gut–heart axis in HFpEF management.

## 1. Introduction

Heart failure with preserved ejection fraction has emerged as the dominant form of heart failure in the 21st century, accounting for over 50% of all heart failure cases with a rising prevalence that parallels global demographic shifts toward aging populations and increasing prevalence of metabolic disorders [1,2]. This complex syndrome is characterized by preserved systolic function (ejection fraction > 50%) but impaired diastolic performance, often accompanied by multiple comorbidities, including obesity, diabetes mellitus, hypertension, and metabolic syndrome. Unlike heart failure with reduced ejection fraction, which has established therapeutic frameworks, HFpEF presents unique pathophysiological challenges that have confounded traditional treatment approaches [3,4]. The syndrome’s heterogeneous nature, coupled with complex interactions between cardiac dysfunction and systemic comorbidities, has led to disappointing results in clinical trials using conventional heart failure therapeutics [5]. This therapeutic gap has intensified the search for novel mechanistic pathways and treatment targets.

This inflammatory burden, often exacerbated by prevalent comorbidities such as obesity, diabetes, hypertension, and chronic kidney disease, initiates a cascade of events leading to endothelial dysfunction, myocardial stiffening, and impaired diastolic function [6]. Given the multisystemic nature of HFpEF, where conditions like diabetes and metabolic syndrome are increasingly recognized as primary drivers, understanding the underlying inflammatory-metabolic phenotype is crucial for developing effective therapeutic strategies [7,8]. Indeed, despite its rising prevalence, HFpEF lacks evidence-based therapies, highlighting a significant unmet need for novel approaches to improve patient outcomes [5,9]. 

The concept of the gut–heart axis has emerged as a paradigm-shifting framework for understanding HFpEF pathogenesis. Recent investigations have revealed significant alterations in gut microbiome composition in HFpEF patients, characterized by reduced microbial diversity, depletion of beneficial bacteria, and enrichment of pro-inflammatory species [10,11]. These changes are associated with altered production of bacterial metabolites, creating a systemic inflammatory milieu that promotes endothelial dysfunction, cardiac remodeling, and diastolic dysfunction. The gut microbiota-inflammation-HFpEF axis represents a bidirectional relationship where gut dysbiosis is associated with cardiovascular dysfunction while cardiac dysfunction further exacerbates intestinal barrier impairment and microbial translocation. This vicious cycle offers novel therapeutic opportunities through microbiome modulation, metabolite replacement therapy, and precision medicine approaches based on individual microbial signatures.

This comprehensive review synthesizes the most recent evidence on the gut–heart axis in HFpEF, with particular emphasis on microbiome-driven inflammation and endothelial dysfunction. We critically evaluate emerging therapeutic strategies and provide a framework for future research directions that may inform future approaches to HFpEF management through precision microbiome medicine. By bridging experimental and clinical evidence, this review proposes gut–heart axis modulation as a promising frontier for innovative HFpEF therapies.

### 1.1. Methodology

This article is a narrative review synthesizing current evidence on the gut–heart axis in HFpEF. Literature was identified through searches of PubMed, MEDLINE, and relevant clinical trial registries using terms including ‘HFpEF,’ ‘gut microbiota,’ ‘TMAO,’ ‘short-chain fatty acids,’ and ‘endothelial dysfunction.’ Given the emerging nature of this field, we included preclinical studies, observational human studies, and available clinical trial data. This narrative approach was chosen due to the heterogeneity of study designs and the hypothesis-generating nature of much of the current evidence. We acknowledge that this review does not follow systematic review methodology, and the conclusions should be interpreted within this context.

### 1.2. Established HFpEF Frameworks

The pathophysiology of HFpEF is now understood to involve multiple interconnected mechanisms. Canonical frameworks include inflammation-driven coronary microvascular dysfunction, which is present in up to 75% of HFpEF patients and is associated with reduced nitric oxide bioavailability, impaired cyclic guanosine monophosphate signaling, and downstream effects on cardiomyocyte relaxation [12,13]. Myocardial stiffening results from both increased collagen deposition and altered titin phosphorylation, contributing to diastolic dysfunction [14]. The systemic comorbidity burden, including obesity, diabetes, hypertension, and chronic kidney disease, creates a proinflammatory milieu that affects not only the heart but also the vasculature, skeletal muscle, and kidneys [13,15]. The gut–heart axis hypothesis should be understood as complementary to, rather than a replacement for, these established frameworks, potentially representing one pathway through which comorbidities exert their effects on cardiac function.

### 1.3. HFpEF Phenotypes and Sex-Specific Considerations

HFpEF represents a heterogeneous syndrome with distinct phenotypic presentations that may have differential relationships with gut microbiome alterations. The obesity-related phenotype, present in over 80% of HFpEF patients, is characterized by excess visceral adiposity, metabolic dysfunction, and systemic inflammation [13,15]. This phenotype may be particularly relevant to gut–heart axis dysfunction given the established links between obesity, gut dysbiosis, and metabolic endotoxemia. The aging-related phenotype involves age-associated changes in body composition, including redistribution of fat to visceral compartments and accumulation of senescent adipose tissue cells [16]. The metabolic HFpEF phenotype encompasses patients with diabetes, insulin resistance, and metabolic syndrome, conditions independently associated with altered gut microbiome composition.

Important sex differences exist in HFpEF pathophysiology. Women have a two-fold-higher lifetime risk of HFpEF compared to HFrEF, and HFpEF is twice as prevalent in women as in men [17]. Women with HFpEF demonstrate more significant concentric LV remodeling, higher diastolic stiffness, and smaller LV chamber size compared to men [18]. The ‘hypertension, arterial stiffness and cardiac remodeling’ and ‘obesity, chronic inflammation and microvascular dysfunction’ phenotypic profiles represent two pathophysiological pathways that are particularly predominant in women [17]. Estrogen provides cardioprotective effects through anti-inflammatory actions on endothelial and immune cells in premenopausal women, protection that may diminish after menopause. These sex-specific differences may influence the relationship between gut microbiome alterations and HFpEF development, though direct evidence in this area remains limited. Future studies should stratify analyses by sex and HFpEF phenotype to better characterize these relationships.

## 2. Gut Microbiome Alterations in HFpEF

Recent investigations into the gut microbiome of patients with heart failure with preserved ejection fraction (HFpEF) have unveiled significant alterations that differentiate these individuals from healthy controls. Notably, a consistent pattern of microbial dysbiosis has been observed, characterized by a decline in beneficial, anti-inflammatory bacterial populations and an increase in potentially pathogenic species. Studies indicate that HFpEF patients display a marked decrease in α-diversity, particularly in species richness. Metrics such as the Chao index show significant reductions in HFpEF patients compared to those without the condition [11,19]. This reduction in microbial diversity has been shown to correlate with disease severity and functional capacity among HFpEF patients [19,20]. 

One of the most critical findings regarding HFpEF-related dysbiosis is the depletion of specific beneficial genera that play vital roles in gut health and inflammatory modulation. Genera such as Butyricicoccus, Lachnospira, and Ruminiclostridium, recognized for their production of short-chain fatty acids (SCFAs) and anti-inflammatory properties, are often reduced in HFpEF patients [11,19]. This loss is thought to contribute to systemic inflammation and cardiac dysfunction, highlighting the pathogenic potential of altered gut microbiota. Concurrently, there is an observed increase in pathogenic microorganisms such as Enterococcus, which can be linked to inflammatory responses [10,19]. 

Importantly, emerging evidence suggests that these microbial shifts in HFpEF patients occur independently of traditional cardiovascular risk factors, such as age, body mass index (BMI), and hypertension, implying that HFpEF itself may uniquely been linked to specific patterns of gut dysbiosis [10,20]. These gut microbiome alterations may warrant consideration in the management and treatment of HFpEF, potentially opening pathways for therapeutic interventions aimed at restoring gut health to improve heart function [10]. 

Metabolomic Consequences:

The observed compositional changes within the gut microbiome profoundly alter the gut metabolome, with significant implications for systemic inflammation and cardiovascular function.

- SCFA Depletion: A direct consequence of the reduction in short-chain fatty acid-producing bacteria is the diminished circulating levels of key SCFAs such as acetate, propionate, and butyrate. This depletion is particularly significant given the established cardioprotective properties of SCFAs and their crucial role in maintaining intestinal barrier integrity [21].
- Harmful Metabolite Accumulation: Patients with HFpEF often exhibit elevated levels of potentially toxic bacterial metabolites. This includes an increase in trimethylamine N-oxide production and altered amino acid metabolism products. These metabolic shifts collectively contribute to a systemic environment characterized by heightened inflammation and pro-fibrotic processes [22].
- Bile Acid Dysregulation: Alterations in gut microbiome composition also lead to a disruption in bile acid metabolism. Such dysregulation has broad implications for both cardiac function and overall metabolic homeostasis. Specifically, certain bile acid profiles, including reduced levels of ursodeoxycholic acid and hyocholic acid, have been associated with an increased risk of HFpEF [23].

## 3. Microbiome-Derived Metabolites: Key Players in HFpEF Pathogenesis

**Figure 1:**
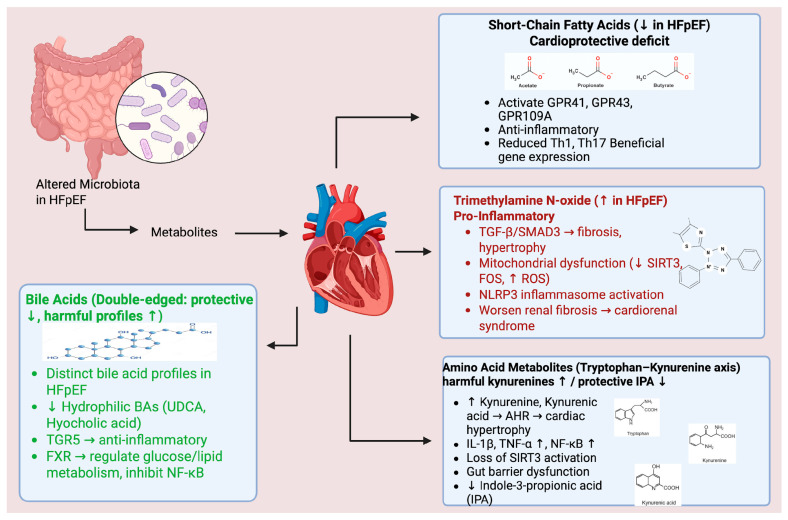
This schematic represents a conceptual model of proposed relationships and does not imply linear causality or established causal pathways.

The observed alterations in gut microbiota composition lead to profound changes in the gut metabolome, generating various metabolites that significantly impact systemic inflammation and cardiovascular function, thereby playing a key role in HFpEF pathogenesis (Figure 1) [24].

### 3.1. Short-Chain Fatty Acids: The Cardioprotective Deficit

Short-chain fatty acids, particularly acetate, propionate, and butyrate, are crucial beneficial metabolites produced by gut microbiota, serving as vital mediators of gut–heart communication. A profound depletion of SCFA-producing bacteria in HFpEF patients may directly contributes to disease pathogenesis. Short-chain fatty acids exert their effects through G protein-coupled receptors known as free fatty acid receptors (FFARs). FFAR3 (GPR41) and FFAR2 (GPR43) respond to acetate, propionate, and butyrate, while GPR109A is specifically activated by butyrate [25,26]. These receptors are expressed on immune cells, intestinal epithelium, and vascular smooth muscle cells [24]. GPR41 and GPR43 play important roles in blood pressure regulation-GPR41 knockout mice are hypertensive, and UK Biobank data show that genetic variants associated with lower GPR41/43 expression are more prevalent in hypertensive individuals [27]. Activation of these receptors triggers anti-inflammatory signaling cascades that inhibit NF-κB-mediated gene expression [24]. Furthermore, SCFAs act as endogenous histone deacetylase inhibitors, promoting beneficial gene expression patterns and reducing inflammatory transcription. Specifically, propionate diminishes NF-κB, IL-6, STAT1, and STAT3 activity, thereby reducing Th1 and Th17 immune responses, while butyrate significantly inhibits NF-κB activity via PPAR-γ stimulation [21,24]. Beyond their anti-inflammatory roles, SCFAs serve as direct cardiac energy substrates and promote beneficial metabolic adaptations, including enhanced branched-chain amino acid catabolism. Experimental studies underscore their importance, demonstrating that SCFA supplementation, especially butyrate, can reverse cardiac dysfunction in HFpEF models. For instance, tributyrin treatment in obese pre-HFpEF mice improved early cardiac dysfunction by upregulating protein phosphatase 2 cm and enhancing branched chain amino acid metabolism [28]. This evidence highlights the SCFA deficit as a clear therapeutic target, with interventions encompassing direct supplementation, prebiotic therapy to promote endogenous production, and dietary modifications to increase fiber intake [19]. 

### 3.2. Trimethylamine N-Oxide: The Inflammatory Culprit

Trimethylamine N-oxide (TMAO) has emerged as a significant pathogenic metabolite in the context of the gut–heart axis, particularly relating to heart failure with preserved ejection fraction (HFpEF). Elevated levels of TMAO have been consistently linked to adverse clinical outcomes in HFpEF patients [29,30,31]. This metabolite is produced by the metabolic activity of gut bacteria, which convert dietary choline and carnitine into trimethylamine (TMA), subsequently oxidized to TMAO in the liver. This illustrates a direct mechanistic relationship between dietary intake, gut microbiome dynamics, and cardiovascular dysfunction [32,33]. 

TMAO may contributes to the pathophysiology of HFpEF through various interconnected pathways. One critical mechanism involves the activation of the TGF-β/SMAD3 signaling pathway, which is implicated in promoting cardiac fibrosis and hypertrophy [34]. Furthermore, TMAO has been shown to induce mitochondrial dysfunction by inhibiting the expression of sirtuin 3 and suppressing the activities of superoxide dismutase 2 and mitochondrial aldehyde dehydrogenase 2, resulting in the accumulation of reactive oxygen species and consequent cellular damage [35]. Additionally, TMAO triggers NLRP3 inflammasome activation, promoting cardiovascular inflammation and endothelial dysfunction, which are pivotal processes in the progression of heart failure [36]. The NLRP3 inflammasome (NACHT, LRR, and PYD domains-containing protein 3) is a multiprotein complex of the innate immune system that senses cellular stress and danger signals [37]. Activation occurs through a two-step process: first, a priming signal (typically via NF-κB activation by toll-like receptor ligands such as lipopolysaccharide) upregulates NLRP3 and pro-IL-1β; second, an activation signal (such as ATP or reactive oxygen species) triggers inflammasome assembly [38,39]. Once assembled, the inflammasome activates caspase-1, which cleaves pro-IL-1β and pro-IL-18 into their mature forms and can induce pyroptosis, a form of inflammatory cell death [37]. In heart failure, NLRP3 inflammasome activation is associated with cardiac hypertrophy, fibrosis, and adverse remodeling, and IL-1β blockade with canakinumab has reduced cardiovascular events in clinical trials [40,41]. 

The implications of elevated TMAO extend beyond direct cardiac effects; it has also been observed to exacerbate renal interstitial fibrosis, thereby accelerating the progression of HFpEF through mechanisms associated with cardiorenal syndrome [30]. Clinically, cross-sectional studies have identified significantly elevated TMAO levels in HFpEF patients compared to healthy controls, underscoring its role as an independent risk factor for negative cardiovascular outcomes. Elevated TMAO correlates with increased mortality and major adverse cardiovascular events and provides valuable stratification in risk assessments, particularly in scenarios where natriuretic peptides do not reflect disease severity accurately [42,43]. 

Ongoing research is focused on exploring multiple strategies to reduce TMAO levels, including dietary modifications, microbiome interventions, and direct inhibition of TMA-producing microbial pathways or the flavin monooxygenase 3 enzyme involved in TMAO synthesis [33]. This multifaceted approach holds promise for mitigating the pro-inflammatory and harm-promoting effects of TMAO, potentially improving the clinical management of HFpEF patients.

#### Conflicting Evidence and Limitations of TMAO Research

While elevated TMAO levels have been associated with adverse cardiovascular outcomes in multiple cohorts, the evidence for a causal relationship remains incomplete and contested [22,44]. Meta-analyses have postulated that high circulating TMAO levels are associated with increased risk of cardiovascular events and all-cause mortality; however, the link between TMAO and CVD is not fully consistent [44]. Importantly, adjustment for renal function tends to decrease or reverse the significant association between TMAO and cardiovascular outcomes, strongly suggesting that the association may be substantially mediated by renal function rather than representing a direct causal pathway [44]. 

Mendelian randomization studies have yielded conflicting results, one study found no significant association between genetically predicted higher TMAO levels and cardiometabolic disease, while another identified a positive causal relationship between TMAO levels and systolic blood pressure [44]. Furthermore, a study in the Framingham Heart Study showed no correlation between plasma TMAO and coronary artery calcium score or carotid intima-media thickness, challenging the direct link between TMAO and atherosclerotic burden [43]. Notably, TMAO levels can be increased by both ‘healthy’ high-fiber diets and ‘unhealthy’ high-choline diets, complicating the interpretation of dietary interventions [43]. 

These findings suggest that TMAO may be a marker of cardiovascular risk rather than a direct causal factor, and its relationship with HFpEF specifically requires further investigation in dedicated studies. The prognostic value of TMAO in HFpEF populations may differ from that observed in coronary artery disease or general cardiovascular cohorts.

### 3.3. Amino Acid Metabolites: The Tryptophan-Kynurenine Axis

Dysregulated amino acid metabolism, particularly the tryptophan-kynurenine pathway, is a significant aspect of the gut–heart axis in heart failure with preserved ejection fraction (HFpEF), representing a potential therapeutic target. Patients with HFpEF have been reported to exhibit increased levels of tryptophan catabolism products such as kynurenine and kynurenic acid, which could be associated with mechanisms like left ventricular remodeling and increased pro-inflammatory cytokines [45]. These metabolites may contribute to HFpEF pathogenesis through several mechanisms, including the activation of the aryl hydrocarbon receptor (AHR), which has been implicated in cardiac hypertrophy [45]. Specifically, kynurenic acid is understood to induce the expression of pro-inflammatory cytokines like IL-1β and TNF-α via AHR signaling.

The aryl hydrocarbon receptor (AHR) is a ligand-activated transcription factor originally identified as a receptor for environmental pollutants but now recognized as a regulator of immune responses and vascular homeostasis [46,47]. AHR is activated by endogenous ligands including tryptophan metabolites produced by gut bacteria (such as indole derivatives) and host enzymes (such as kynurenine) [48]. Upon ligand binding, AHR translocates to the nucleus and induces expression of downstream genes including cytochrome P450 enzymes [49]. A systematic review concluded that AHR activation leads to vascular oxidative stress and endothelial dysfunction, and blocking AHR signaling could provide a therapeutic target for vascular disorders [48]. Recent studies demonstrate that whole-body AHR deletion ameliorates atherosclerosis through altered lipid metabolism [50]. 

Conversely, indole-3-propionic acid (IPA), a beneficial metabolite derived from tryptophan, is observed to be significantly reduced in HFpEF patients [51]. IPA has illustrated therapeutic potential by activating SIRT3, thus enhancing the NAD+ pathway and improving gut barrier integrity [52]. Moreover, kynurenine pathway metabolites are of growing interest as potential biomarkers for the diagnosis and prognosis of HFpEF. Therapeutic approaches aimed at modulating tryptophan metabolism, which include IPA supplementation and inhibition of the kynurenine pathway, are currently under investigation to explore their potential utility in HFpEF management [52]. 

### 3.4. Bile Acids: The Double-Edged Metabolites

Bile acids are complex signaling molecules that exert both beneficial and detrimental effects on cardiovascular health. Recent research has identified specific bile acid profiles associated with HFpEF risk and therapeutic potential. HFpEF patients, particularly those with metabolic dysfunction-associated fatty liver disease, exhibit distinct bile acid signatures, including significantly lower levels of ursodeoxycholic acid and hyocholic acid species [53,54]. 

Hydrophilic bile acids, including ursodeoxycholic acid, may confer protective effects on cardiac tissues through mechanisms such as TGR5 receptor activation. Activation of TGR5 has been linked to anti-inflammatory signaling pathways that support cardiac function and mitigate inflammation [55]. TGR5 (Takeda G protein-coupled receptor 5, also known as GPBAR1) is a membrane-bound receptor activated by secondary bile acids, particularly lithocholic acid [56]. Unlike FXR, TGR5 signals through the cell membrane by increasing intracellular cyclic AMP levels [57]. TGR5 is expressed in endothelial cells, macrophages, and vascular smooth muscle [58]. In endothelial cells, TGR5 activation induces nitric oxide production via Akt phosphorylation, promoting vasodilation and reducing monocyte adhesion [59]. TGR5 also exerts anti-inflammatory effects in macrophages by inhibiting NF-κB activity, which has been shown to attenuate atherosclerosis in animal models [58]. 

Additionally, bile acids also engage the FXR pathway, playing a significant role in regulating glucose and lipid metabolism while inhibiting NF-κB signaling, which reduces inflammatory responses [60]. The farnesoid X receptor (FXR, also known as NR1H4) is a nuclear receptor activated by primary bile acids, particularly chenodeoxycholic acid [61]. Upon activation, FXR regulates genes involved in bile acid synthesis, glucose metabolism, and lipid homeostasis [62]. FXR is expressed in the liver, intestine, and cardiovascular tissues including vascular smooth muscle cells and macrophages, where it influences cholesterol transport and reverse cholesterol transport from foam cells [63]. FXR activation inhibits NF-κB signaling, reducing inflammatory responses, and FXR agonists have demonstrated anti-atherosclerotic effects in preclinical models [64,65]. 

The therapeutic potential of bile acids is under investigation, with strategies focusing on bile acid receptor agonists and supplementation with cardioprotective bile acids, such as taurine-conjugated bile acids, showing promise in experimental models of HFpEF [66]. Emerging evidence also indicates that bile acid signaling may be leveraged to improve metabolic outcomes in HFpEF patients, suggesting that interventions designed to modulate bile acid metabolism could represent a novel therapeutic approach in the management of HFpEF [67]. 

## 4. Endothelial Dysfunction in the Gut–Heart Axis

Endothelial dysfunction represents a critical pathophysiological component of HFpEF, with gut microbiome alterations serving as a major driver of this impairment. The gut–heart axis may contributes to endothelial dysfunction through multiple interconnected pathways that ultimately compromise coronary microvascular function and promote diastolic dysfunction.

Mechanisms of Microbiome-Mediated Endothelial Impairment.

Microbiome-derived factors contribute significantly to endothelial damage:

- TMAO-Induced Endothelial Damage: Trimethylamine N-oxide directly impairs endothelial function through several mechanisms. It enhances endothelial cell apoptosis and inflammatory activation, promotes oxidative stress, and reduces nitric oxide bioavailability. Furthermore, TMAO facilitates foam cell formation, accelerates atherosclerosis, and impairs endothelium-dependent vasodilation, all contributing to endothelial dysfunction [68].
- LPS-Mediated Endothelial Activation: Bacterial lipopolysaccharides, crossing the compromised gut barrier, activate endothelial cells via Toll-like receptor 4 signaling. This activation leads to the upregulation of adhesion molecules (ICAM-1, VCAM-1) and chemokines, increasing vascular permeability and inflammatory cell recruitment. This process ultimately contributes to a pro-thrombotic endothelial phenotype and activates complement and coagulation cascades [69,70,71].
- SCFA Deficiency Effects: A reduction in beneficial short-chain fatty acid levels may also contributes to endothelial dysfunction. This deficiency results in decreased G-protein coupled receptor-mediated protective signaling in endothelial cells, impairs endothelial barrier function leading to increased permeability, and reduces the production of anti-inflammatory mediators. Consequently, it compromises endothelial repair and regeneration mechanisms, exacerbating dysfunction [72,73].

### 4.1. Zonulin and Intestinal Barrier Dysfunction

Zonulin, the only known physiological modulator of intestinal tight junctions, has emerged as a potentially important mediator in the gut–heart axis [74]. Elevated plasma zonulin levels have been observed in patients with chronic heart failure and correlate with cardiac dysfunction, including higher left ventricular end-systolic dimension and lower ejection fraction [75]. Zonulin regulates intestinal permeability by disassembling tight junctions, and its dysregulation may facilitate bacterial translocation and metabolic endotoxemia [74,76]. In coronary artery disease patients, elevated zonulin levels are associated with increased intestinal permeability and detection of bacterial 16S rRNA in blood samples [76]. Recent longitudinal data suggest that zonulin, along with lipopolysaccharide-binding protein, predicts cardiovascular mortality and correlates with conventional cardiovascular risk factors [77]. Zonulin antagonists, such as larazotide acetate, have shown promise in improving gut barrier integrity in other inflammatory conditions and may represent a therapeutic target warranting investigation in HFpEF [78]. However, direct evidence linking zonulin to HFpEF specifically remains limited, and this represents an important area for future research.

### 4.2. Clinical Implications of Endothelial Dysfunction

The clinical implications of endothelial dysfunction, particularly in the context of heart failure with preserved ejection fraction (HFpEF), are significant, impacting patient management and therapeutic strategies. Endothelial dysfunction has been associated with coronary microvascular dysfunction, exercise intolerance, and the overall progression of heart failure pathology. Understanding these implications reinforces the need for targeted therapeutic interventions aimed at these mechanisms.

Endothelial dysfunction severely compromises coronary microvascular function, leading to impaired coronary flow reserve and increased microvascular resistance, which restricts oxygen delivery to cardiac tissues [79]. These factors are central to the pathophysiology of HFpEF and contribute to exercise intolerance in affected patients. The inadequate ability of both peripheral and coronary vasculature to dilate during physical activity exacerbates hemodynamic instability and diminishes exercise capacity, ultimately impairing quality of life [79]. Furthermore, chronic endothelial dysfunction can accelerate the progression of HFpEF, resulting in increased arterial stiffness, cardiac remodeling, and potential onset of cardiorenal syndrome, thereby worsening patient outcomes [72,79]. 

Given these challenges, several therapeutic strategies targeting endothelial dysfunction present opportunities for improving patient outcomes. For instance, restoring gut microbiome balance through approaches such as fecal microbiota transplantation (FMT) or the administration of probiotics can enhance endothelial function by decreasing harmful metabolite production and promoting beneficial microbial profiles [80]. Additionally, direct interventions focusing on short-chain fatty acids (SCFAs) supplementation and strategies aimed at reducing trimethylamine N-oxide (TMAO) levels may positively influence endothelial integrity and cardiovascular health [79]. Anti-inflammatory strategies, including modulation of the NLRP3 inflammasome and NF-κB pathways, may offer additional avenues to counteract the pro-inflammatory state associated with gut-derived endotoxemia and endothelial dysfunction [79,81]. 

The complex nature of the gut–heart axis emphasizes the urgent need for comprehensive strategies to address endothelial dysfunction in HFpEF patients. By implementing microbiome-focused interventions, healthcare professionals may improve patient outcomes and better manage this intricate condition.

## 5. Therapeutic Interventions Targeting the Gut–Heart Axis in HFpEF

### 5.1. Fecal Microbiota Transplantation: Comprehensive Microbiome Restoration

FMT represents a comprehensive approach to gut microbiome restoration, offering significant potential for fundamentally correcting the dysbiosis underlying HFpEF. Recent experimental evidence strongly supports its therapeutic promise. Preclinical studies, such as those conducted in obesity-associated pre-HFpEF mouse models, have demonstrated remarkable therapeutic effects of FMT from lean donors. These effects include improvement in systolic and diastolic function, along with reductions in cardiac hypertrophy and pathological remodeling [82]. These outcomes are thought to be linked to an increase in butyrate-producing bacteria, highlighting the microbiome’s role in cardiovascular health [83]. Moreover, investigations into diet-induced dysbiosis reveal that FMT can improve metabolic health, suggesting that gastrointestinal health restoration may yield significant cardiovascular benefits [84]. Safety considerations are paramount when considering FMT translation to cardiovascular populations. Serious adverse events, including transmission of multidrug-resistant organisms, have been reported, with the FDA issuing safety alerts regarding extended-spectrum β-lactamase-producing Escherichia coli transmission [85,86]. FMT-related serious adverse events occur in approximately 1.4% of procedures, with higher risk in immunocompromised patients and those with mucosal barrier injury [87]. Long-term safety data remain limited, and the potential for durable transmission of microbial signatures that could affect disease risk is unknown [88]. Regulatory barriers present additional challenges. FMT is considered investigational by most regulatory agencies, with variable classification across countries [88,89]. In the United States, the FDA maintains enforcement discretion for recurrent Clostridioides difficile infection but requires investigational new drug applications for other indications. The standardization of donor selection, microbiome characterization, and delivery protocols remains incomplete [85,88]. 

Translational challenges: The translation of FMT from preclinical success to clinical application faces substantial hurdles [90,91,92]. Animal models of HFpEF, while valuable for mechanistic insights, do not fully recapitulate the heterogeneity, comorbidity burden, and polypharmacy characteristic of human HFpEF populations [91]. A consensus statement from the Human Microbiome Action Consortium emphasized that preclinical models offer crucial insights but determining underlying mechanisms and establishing cause and effect is extremely difficult [91]. Interventions such as FMT often yield inconsistent or modest effects in clinical trials compared to preclinical studies, highlighting the need for precision approaches and functional profiling [93]. The field has faced a deluge of correlative ‘dysbiosis’ studies with limited causal evidence, and rigorous iterative testing from proof-of-concept experiments to deep mechanistic understanding is required before clinical translation [90]. 

Given these limitations, FMT should be considered a hypothesis-generating experimental approach rather than an emerging clinical strategy for HFpEF. Rigorous clinical trials with appropriate safety monitoring will be essential before any clinical application can be considered.

### 5.2. Probiotic Interventions: Targeted Microbial Supplementation

Probiotic interventions present a targeted strategy for microbiome modulation, particularly with promising implications for heart failure with preserved ejection fraction (HFpEF) populations. Ongoing clinical studies are exploring the efficacy of specific probiotic strains on metabolic improvements and heart failure outcomes. Notably, the trial titled “Influence of Probiotics on Metabolome and Heart Failure” is investigating the effects of Lactobacillus rhamnosus supplementation, which has been associated with changes in metabolic profiles and beneficial cardiac function biomarkers [94]. This emphasis on specific strains highlights the need for tailored probiotic applications to enhance therapeutic outcomes in HFpEF.

Advanced formulations combining multiple probiotic strains are also being researched for their potential synergistic effects on inflammation and cardiovascular function [95,96]. Evidence suggests that probiotics may address HFpEF outcomes through a multitude of mechanisms, including restoring microbial diversity, enhancing the production of beneficial metabolites, and competitively excluding pathogenic bacteria all of which can positively influence gut health [97,98]. Probiotics are especially promising in reducing trimethylamine N-oxide (TMAO) synthesis, which is linked to cardiovascular risk, as well as enhancing gut barrier function to mitigate bacterial translocation into circulation [98,99]. 

The direct anti-inflammatory effects of probiotics, facilitated by immune modulation, further underline their therapeutic relevance in managing HFpEF-related pathophysiology [92,100]. As ongoing research continues to unravel the complexities of probiotics in cardiovascular health, their application in clinical settings appears increasingly viable. The findings from these investigations may pave the way for refined probiotic formulations that can be strategically integrated into treatment regimens for patients managing HFpEF.

### 5.3. Metabolite-Based Therapies and Precision Medicine Approach

Direct supplementation of beneficial metabolites or inhibition of harmful metabolite production represents a precision medicine approach to targeting the gut–heart axis, particularly in heart failure with preserved ejection fraction (HFpEF). Emerging studies have highlighted the potential of short-chain fatty acid (SCFA) supplementation in HFpEF models. Specifically, tributyrin, a butyrate prodrug, has been found to improve cardiac function and reduce fibrosis [101]. Additionally, sodium butyrate supplementation has enhanced cardiac energy metabolism, and propionate supplementation has been shown to reduce systemic inflammation and myocardial fibrosis [102]. 

Indole-3-propionic acid (IPA) is gaining attention as a therapeutic target with potential benefits for HFpEF. Its mechanisms include activation of SIRT3, enhancement of NAD+ pathways, reduction in oxidative stress and inflammation, and improvement of gut barrier function and microbiome composition. These processes collectively support its cardioprotective effects through metabolic modulation, positioning IPA as a beneficial agent in managing HFpEF.

In parallel, strategies aimed at reducing harmful trimethylamine N-oxide (TMAO) levels are being investigated. Approaches include dietary modifications that lower choline and carnitine intake, microbiome modulation to decrease TMA-producing bacteria, and inhibition of the flavin monooxygenase 3 (FMO3) enzyme, crucial in TMAO synthesis [103,104]. The development of TMAO-binding compounds for enhanced elimination is also in progress, indicating a multifaceted strategy to mitigate cardiovascular risks associated with TMAO [105]. 

Looking ahead, deploying precision medicine approaches tailored to individual microbiome responses and metabolic profiles is crucial. Ongoing efforts to create biomarker panels incorporating gut microbiome-derived metabolites aim to facilitate early detection of HFpEF, risk stratification, and personalized intervention strategies [106]. The integration of multi-omics data comprising genomics, metabolomics, and microbiomics promises to identify novel therapeutic targets and optimize combination therapies for individual patient needs [107]. Furthermore, advancements in digital health technologies, such as wearable devices integrated with microbiome analysis, will enable real-time monitoring of therapeutic responses, enhancing personalized dietary recommendations and optimizing intervention timing [108]. Therefore, precision medicine approaches focusing on metabolic and microbiome modulation concerning the gut–heart axis hold promise for improving outcomes in patients with HFpEF.

## 6. Future Directions and Research Opportunities

The gut–heart axis in HFpEF presents numerous unexplored avenues for research, offering transformative opportunities that could revolutionize treatment approaches and patient outcomes (Table 1).

### 6.1. Novel Therapeutic Targets

Beyond conventional approaches, several novel therapeutic targets are emerging from research into the gut–heart axis. Recent evidence suggests that Paneth cell dysfunction contributes significantly to gut–heart axis disruption. Paneth cells, specialized intestinal epithelial cells that produce antimicrobial peptides, may represent a novel therapeutic target for maintaining gut barrier function and preventing bacterial translocation [109,110]. Recent studies have elucidated the impact of Paneth cell dysfunction in conditions like obesity and inflammatory bowel disease (IBD), highlighting their role in regulating gut microbiota and preventing dysbiosis [111,112,113]. For instance, a report noted that bile acid toxicity within Paneth cells can exacerbate dysbiosis, suggesting a potential target for therapeutic intervention [114]. Additionally, research indicates that supplementation of products derived from Paneth cells, such as defensins, can restore gut microbiota balance after dysregulation [109]. 

Understanding microbiome–drug interactions represents another crucial aspect of optimizing therapies for heart failure with preserved ejection fraction (HFpEF). Current medications, particularly SGLT2 inhibitors, show promise not only for their cardioprotective effects but also for their ability to modulate gut microbiome composition, which may contribute to their therapeutic efficacy [115,116]. The complex relationship between medications and the gut microbiome necessitates further exploration to optimize treatment strategies effectively. Additionally, integrating circadian rhythm research offers a unique perspective on gut–heart signaling, given that the gut microbiome exhibits circadian rhythms that can profoundly influence cardiovascular function. Chronotherapy approaches that consider the timing of interventions may enhance therapeutic outcomes for HFpEF patients [117]. 

Moreover, phage therapy is emerging as a precision medicine strategy to modulate the gut microbiome selectively. Phage therapy’s ability to target specific pathogenic bacteria while preserving beneficial microbial populations could lead to personalized treatments that mitigate dysbiosis-related issues in HFpEF patients [110,118]. 

### 6.2. Advanced Diagnostic Approaches

The development of advanced diagnostic tools is essential for harnessing the full potential of gut–heart axis research in heart failure with preserved ejection fraction (HFpEF). Metabolomic profiling, combined with comprehensive clinical assessments, can facilitate the early identification of HFpEF risk in asymptomatic individuals and monitor treatment efficacy through metabolite tracking [119,120]. This advancement may foster the creation of point-of-care diagnostic tools that enhance patient management in clinical settings.

Artificial intelligence applications to multi-omics datasets integrating genomics, metabolomics, and microbiomics hold promise for identifying novel biomarker combinations that could improve diagnostic accuracy. These approaches may aid in predicting optimal therapeutic interventions for individual patients, discovering previously unrecognized pathogenic pathways, and enabling real-time treatment optimization based on continuous monitoring [121,122,123]. For instance, systems utilizing AI to analyze extensive omics data can uncover complex interactions that might otherwise remain hidden, thus refining treatment strategies and improving outcomes for HFpEF patients [124]. 

Moreover, the integration of advanced cardiac imaging techniques with microbiome analysis could yield valuable insights into correlations between microbiome signatures and specific cardiac phenotypes. This dual approach may facilitate the identification of subclinical disease progression markers and guide imaging-informed microbiome interventions [120]. 

### 6.3. Therapeutic Innovation

Innovative therapeutic strategies are being explored to directly target the gut–heart axis in HFpEF. Advances in gene therapy, such as trials involving SERCA2a gene therapy for HFpEF, could be further enhanced through combinations with microbiome interventions focused on inflammation and metabolism [125,126]. Regenerative medicine approaches, including stem cell therapies and tissue engineering, could significantly benefit from concomitant microbiome optimization, creating favorable inflammatory environments conducive to cardiac repair and regeneration.

Rational design of combination therapies is also crucial, which includes integrating traditional HFpEF medications with microbiome modulators, combining multiple microbiome-targeting approaches for synergistic effects, and incorporating dietary interventions alongside pharmacological microbiome modulation [127,128]. Furthermore, the exploration of the integration of device-based therapies with microbiome optimization represents an exciting frontier in therapeutic innovation [129]. Such multi-faceted strategies are likely to enhance the efficacy of existing treatments while paving the way for novel interventions tailored to the unique needs of HFpEF patients.

### 6.4. Population Health Implications

Understanding the gut–heart axis carries significant implications for population health, enabling novel prevention strategies through early interventions tailored to high-risk individuals based on their microbiome signatures. This knowledge fosters public health initiatives promoting gut microbiome health and the development of dietary guidelines optimized for cardiovascular benefits. Research indicates that diets high in fiber, which are essential for gut health, can lower the risk of cardiovascular diseases. For example, studies have shown that dietary patterns such as the Dietary Approaches to Stop Hypertension (DASH) and Mediterranean diets significantly improve gut health and subsequently cardiovascular outcomes [105]. 

Furthermore, public health efforts must consider environmental interventions aimed at reducing exposures that disrupt the microbiome. For instance, the Dutch Microbiome Project has highlighted various factors that shape a healthy gut microbiome, emphasizing the importance of maintaining these conditions to support public health. Additionally, understanding how social determinants of health influence the gut–heart axis is essential for addressing health disparities. Variations in microbiome patterns have been observed across different populations, influenced by access to microbiome-supporting foods and interventions [130]. Cultural factors and economic barriers also play significant roles in dietary patterns and gut health, underscoring the need to consider these factors in public health strategies [131]. 

As evidenced by recent research, there are intrinsic relationships between gut microbiota composition and chronic health conditions such as hypertension, which shows variations based on dietary and lifestyle changes across populations [132]. This reinforces the potential of leveraging microbiome insights into effective public health strategies tailored to specific demographic groups to optimize cardiovascular health on a larger scale [133]. 

Moreover, ongoing studies investigate the impact of maternal diet and the consequent gut microbiota composition in offspring, which may predispose them to cardiovascular diseases later in life [134]. This longitudinal perspective highlights the importance of dietary interventions that foster healthy gut microbiomes from early stages to mitigate future health risks.

## 7. Methodological Considerations and Limitations of Current Evidence

Several important limitations should be acknowledged when interpreting the evidence presented in this review. The majority of mechanistic evidence derives from preclinical animal models, which may not fully recapitulate human HFpEF pathophysiology. Human studies are predominantly cross-sectional and observational, precluding causal inference regarding whether microbiome alterations precede, accompany, or result from cardiac dysfunction. Longitudinal studies with serial sampling are essential to establish temporal relationships.

HFpEF heterogeneity presents a significant challenge, as the syndrome encompasses multiple phenotypes with potentially distinct pathophysiological mechanisms. Most studies have not stratified by HFpEF phenotype, sex, or comorbidity burden. Methodological heterogeneity further complicates interpretation: most studies employ 16S rRNA gene sequencing, which has lower taxonomic resolution and produces sparser data compared to shotgun metagenomics [135,136,137,138]. Differences in sequencing platforms, bioinformatic pipelines, and sample handling limit comparability and generalizability across studies.

Multiple confounding factors remain inadequately addressed. Diet is a major determinant of gut microbiome composition and metabolite production (e.g., TMAO levels), yet dietary assessment is often limited or absent [43]. Common cardiovascular medications including proton pump inhibitors, metformin, statins, and antibiotics significantly alter gut microbiome composition [90,93], but polypharmacy effects are poorly characterized and studies rarely account for medication-microbiome interactions. Additionally, obesity, diabetes, and other HFpEF-associated comorbidities independently affect gut microbiome composition, making it difficult to isolate HFpEF-specific effects. The gut microbiome also exhibits substantial inter-individual variability influenced by genetics, geography, diet, age, and environmental exposures [90,139], limiting generalizability of single-center studies.

Finally, translational uncertainty remains substantial. No randomized controlled trials have evaluated microbiome-targeted interventions specifically in HFpEF populations. The therapeutic sections of this review should be interpreted as identifying potential future research directions rather than near-term clinical strategies, as safety, feasibility, and regulatory considerations require careful evaluation before clinical application.

## 8. Conclusions

Established Mechanisms: Gut microbiota dysbiosis is consistently observed in heart failure populations, characterized by reduced microbial diversity and altered metabolite profiles. SCFAs exert anti-inflammatory and cardioprotective effects through well-characterized receptor-mediated pathways (GPR41/43/109A). TMAO is associated with adverse cardiovascular outcomes in multiple cohorts, though causality remains debated. Systemic inflammation, mediated in part through LPS translocation and NLRP3 inflammasome activation, contributes to endothelial dysfunction and cardiac remodeling.

Emerging but Incomplete Evidence: Specific gut microbiome signatures may distinguish HFpEF patients from healthy controls, though studies are limited by small sample sizes and cross-sectional designs. Preclinical studies suggest therapeutic potential for FMT, SCFA supplementation, and probiotic interventions, but human data in HFpEF are lacking. The bidirectional nature of the gut–heart axis, whereby cardiac dysfunction may exacerbate intestinal barrier impairment, offers multiple potential intervention points but requires longitudinal validation.

Speculative and Future Concepts: Precision microbiome medicine, AI-guided therapy optimization, and combination microbiome-targeted therapies represent promising but currently hypothetical approaches. Large-scale clinical trials specifically targeting HFpEF populations are needed before any microbiome-based intervention can be recommended for clinical use.

The gut–heart axis represents a compelling framework for understanding HFpEF pathophysiology and identifying novel therapeutic targets. However, the field must transition from associative observations to causal mechanistic studies and rigorous clinical trials before these insights can be translated into improved patient outcomes.

## Figures and Tables

**Table 1:** Hypothetical Research Concepts for Future Investigation (Note: These represent proposed study designs to address evidence gaps identified in this review. They do not describe ongoing or completed clinical trials.).

Proposed Study (Acronym)	Objective	Proposed Design	Primary Endpoints	Secondary Endpoints	Key Innovation
**Precision Microbiome Medicine for HFpEF**	Develop and validate a precision medicine platform for personalized microbiome interventions in HFpEF patients	Multi-center RCT; personalized interventions vs. standard care; n = 1000	Composite CV outcomes, QoL, exercise capacity	Microbiome restoration, metabolite profiles, inflammatory biomarkers	ML-guided real-time microbiome monitoring for treatment optimization
**Gut–Heart Metabolite Intervention Trial**	Assess efficacy/safety of combined SCFA + IPA replacement vs. placebo in metabolite-deficient HFpEF	Phase II/III double-blind RCT; n = 500	Peak VO_2_, NT-proBNP, diastolic function	Endothelial function, inflammation, gut barrier integrity	First precision metabolite replacement therapy trial in CVD
**Early HFpEF Detection Through Microbiome Analysis**	Develop microbiome biomarkers for early HFpEF detection in at-risk individuals	Prospective cohort; 5-year follow-up; n = 2000	Time to HFpEF diagnosis, predictive accuracy	Intermediate phenotypes, cost-effectiveness	First large-scale microbiome-based CV prediction study
**Microbiome Targeted Combination Therapy**	Evaluate synergy of FMT, probiotics, and metabolite supplementation in refractory HFpEF	Adaptive randomized trial; n = 300	Clinical improvement, HF hospitalization rates, QoL	Microbiome restoration, safety, biomarkers	First systematic combination microbiome therapy trial in HFpEF
**Pediatric-to-Adult Microbiome Trajectory Study**	Determine early-life microbiome influence on adult HFpEF risk	20-year longitudinal cohort; n = 1000	Adult CV risk profile, HFpEF incidence	Metabolic health, inflammatory profiles, intervention windows	First lifespan microbiome–cardiovascular link study
**Environmental Modulation of the Gut–Heart Axis**	Investigate environmental factors affecting gut–heart axis and HFpEF risk	Multi-cohort; varied exposures; n = 5000	Environmental associations with microbiome disruption and HFpEF	Mechanistic pathways, reversibility	First comprehensive study of environmental determinants of CV-relevant microbiome changes
**Artificial Intelligence-Guided Microbiome Therapy**	Develop AI algorithms for real-time microbiome therapy optimization	Single-arm pilot; wearable and microbiome monitoring; n = 100	Feasibility, clinical improvement	Algorithm performance, patient acceptability, cost-effectiveness	First AI-driven real-time microbiome therapy approach in CVD
**Microbiome–Heart Device Integration Study**	Test whether microbiome optimization enhances device-based HFpEF therapy efficacy	RCT: device therapy vs. device + microbiome optimization; n = 200	Device efficacy enhancement, clinical outcomes	Inflammatory profiles, device complications, mechanisms	First integration of microbiome therapy with device-based HFpEF treatment

## Data Availability

Data sharing is not applicable. No new data were created or analyzed in this study.
